# Upregulation of PBP1B and LpoB in *cysB* Mutants Confers Mecillinam (Amdinocillin) Resistance in Escherichia coli

**DOI:** 10.1128/AAC.00612-19

**Published:** 2019-09-23

**Authors:** Elisabeth Thulin, Dan I. Andersson

**Affiliations:** a Department of Medical Biochemistry and Microbiology, Uppsala University, Uppsala, Sweden

**Keywords:** *Escherichia coli*, amdinocillin, antibiotic resistance, cell wall, *cysB*, cysteine, *ftsZ*, *lpoB*, mechanisms of resistance, mecillinam, penicillin-binding protein, redox state

## Abstract

Mecillinam (amdinocillin) is a β-lactam antibiotic that inhibits the essential penicillin-binding protein 2 (PBP2). In clinical isolates of Escherichia coli from urinary tract infections, inactivation of the *cysB* gene (which encodes the main regulator of cysteine biosynthesis, CysB) is the major cause of resistance. How a nonfunctional CysB protein confers resistance is unknown, however, and in this study we wanted to examine the mechanism of resistance. Results show that *cysB* mutations cause a gene regulatory response that changes the expression of ∼450 genes. Among the proteins that show increased levels are the PBP1B, LpoB, and FtsZ proteins, which are known to be involved in peptidoglycan biosynthesis. Artificial overexpression of either PBP1B or LpoB in a wild-type E. coli strain conferred mecillinam resistance; conversely, inactivation of either the *mrcB* gene (which encodes PBP1B) or the *lpoB* gene (which encodes the PBP1B activator LpoB) made *cysB* mutants susceptible. These results show that expression of the proteins PBP1B and LpoB is both necessary and sufficient to confer mecillinam resistance. The addition of reducing agents to a *cysB* mutant converted it to full susceptibility, with associated downregulation of PBP1B, LpoB, and FtsZ. We propose a model in which *cysB* mutants confer mecillinam resistance by inducing a response that causes upregulation of the PBP1B and LpoB proteins. The higher levels of these two proteins can then rescue cells with mecillinam-inhibited PBP2. Our results also show how resistance can be modulated by external conditions such as reducing agents.

## Abstract (precis)

Mecillinam (amdinocillin) is a β-lactam antibiotic that inhibits the essential penicillin-binding protein 2 (PBP2). In clinical isolates of Escherichia coli from urinary tract infections, inactivation of the *cysB* gene (which encodes the main regulator of cysteine biosynthesis, CysB) is the major cause of resistance. How a nonfunctional CysB protein confers resistance is unknown, however, and in this study we wanted to examine the mechanism of resistance.

## INTRODUCTION

β-Lactams constitute the clinically most important antibiotic group, being prescribed more than all other antibiotics combined (1). Their common functional structure, the β-lactam ring, enables them to bind penicillin-binding proteins (PBPs). PBPs are responsible for the synthesis and maintenance of peptidoglycan (PG), the essential structure that forms the bacterial cell wall, which makes them an ideal target for antibiotics. Escherichia coli has 11 PBPs, with different transpeptidase (TPase), carboxypeptidase, or endopeptidase activities (2). The PBPs are divided into classes A, B, and C on the basis of their enzymatic activities. Mecillinam (amdinocillin) is a penicillin-type β-lactam that is used exclusively for the treatment of urinary tract infections (UTIs) (3–5). Mecillinam specifically targets PBP2 (6–8), which is an essential protein that is part of the elongasome (Rod system). The Rod system is a complex composed of a number of proteins, including the actin-like MreB and its associated proteins MreC, MreD, RodZ, RodA, and PBP2 (2). The elongasome is involved during the elongation mode of PG synthesis in many rod-shaped bacteria (2). When bacteria are treated with mecillinam, they become large spheres and eventually lyse (8, 9).

Mutations in >100 genes can confer mecillinam resistance (Mec^r^); as a result, resistant mutants are easily selectable in the laboratory (10–20). Importantly, in our previous study of Mec^r^ in a collection of clinical E. coli isolates from UTIs, mutations in the *cysB* gene were the major cause of resistance (11). Loss-of-function mutations in *cysB* that conferred Mec^r^ were first described in Salmonella enterica serovar Typhimurium by Oppezzo and Antón (12).

Many of the known Mec^r^ mutations result in increased intracellular levels of the stringent response signal molecule ppGpp (13–16), but this is not the case for *cysB* mutations. As shown by Costa and Antón, *cysB* mutants remain Mec^r^ when the ppGpp synthetase genes *relA* and *spoT* are deleted (1, 21).

The ppGpp-mediated resistance is dependent on increases in the cell division protein FtsZ, and the increases in FtsZ render the Rod system nonessential. However, overproduction of FtsZ alone cannot confer Mec^r^ (2, 16). More recent results show that mecillinam has a dual cellular effect, inhibiting the TPase activity of PBP2 and also causing the activity of the Rod system to become toxic, due to a futile cycle of PG synthesis and degradation performed by the Rod complex and Slt (lytic transglycosylase) (3–5, 22). The futile cycle of formation of un-cross-linked glycans and rapid degradation by Slt is not specific to mecillinam but seems to be a common feature among β-lactams (6–8, 22). This finding suggests that, by inactivating the Rod system and rendering PBP2 nonessential for growth, a strain could become Mec^r^.

Because our previous study showed that *cysB* mutations represented the major cause of Mec^r^ in clinical isolates, we wanted to elucidate the mechanism of Mec^r^ in *cysB* mutants (2, 11). The CysB protein is the major transcriptional regulator of genes encoding proteins involved in sulfur metabolism (including cysteine biosynthesis), positively regulating a number of *cys* operons (2, 23). Cysteine is a key molecule in the cell, not only as a building block in proteins but also as a redox-sensitive molecule. For example, the l-cysteine/l-cystine shuttle system is located in the inner membrane of E. coli and provides reducing equivalents to the periplasm (8, 9, 24). In addition, cysteine residues in proteins not only can form disulfide bonds but also are sites that can have several redox states, which can change the enzymatic activity or binding activity of the protein (10–20, 25). In this study, we show that the mecillinam resistance of *cysB* mutants is conferred by upregulation of the PBP1B and LpoB proteins, which can bypass the need for PBP2, and that reducing agents can reverse this upregulation and the resistance phenotype.

## RESULTS

### Functional LpoB and PBP1B proteins are needed for mecillinam resistance.

We hypothesized that the (over)expression of a PBP other than the mecillinam target PBP2 could be the cause of Mec^r^ in *cysB* mutants. To test this, we inactivated the genes encoding all nonessential PBPs and β-lactam-interacting proteins in the Δ*cysB* mutant by insertion of a Kan cassette from the Keio collection and determined the level of resistance in the constructed mutants.

The Δ*cysB mrcB*::Kan (strain DA49479) and Δ*cysB lpoB*::Kan (strain DA50858) mutants both became fully susceptible (MIC, 0.125 mg/liter), compared to the Δ*cysB* mutant (MIC, 32 mg/liter), clearly demonstrating that the functions of both of these proteins are needed to confer resistance in the Δ*cysB* mutant (Table 1). The inactivation of PBP1A (*mrcA*), PBP1C (*pbpC*), PBP4 (*dacB*), PBP4B (*yfeW*), PBP5 (*dacA*), PBP6A (*dacC*), PBP6B (*dacD*), AmpC (*ampC*), and AmpH (*ampH*) had no effect on resistance (see Table S3 in the supplemental material), demonstrating that it is specifically PBP1B (MrcB) and LpoB that are essential for Mec^r^.

**TABLE 1:** MICs of mecillinam for the wild-type (DA5438), Δ*cysB* (DA28439), *lpoB* duplication (DA55581), *mrcB* duplication (DA58001), Δ*cysB lpoB*::Kan (DA50858), and Δ*cysB mrcB*::Kan (DA49479) strains on MHA without and with cysteine

E. coli strain	Genotype	Mecillinam MIC (mg/liter)	
		MHA	MHA + Cys
DA5438	Wild type	0.125	0.19
DA28439	Δ*cysB*	32	0.38
DA55581	*lpoB* duplication	24	12
DA58001	*mrcB* duplication	1	1
DA50858	Δ*cysB lpoB*::Kan	0.125	ND^*a*^
DA49479	Δ*cysB mrcB*::Kan	0.125	ND

a ND, not determined.

To examine whether inactivating the *lpoB* or *mrcB* gene had the general effect of reducing the MIC of β-lactams, the MICs of meropenem, cefotaxime, and ampicillin were determined for the Δ*lpoB* (strain K3194) and Δ*mrcB* (strain K303) mutants and the parental wild-type strain DA28696 (Table S4). The MICs of these β-lactams were not reduced significantly by inactivation of *lpoB* or *mrcB*, demonstrating that the effect is specific for mecillinam. Similarly, deletion of *cysB* (DA28439) did not change the susceptibility to any of the β-lactams listed above (Table S4).

### CysB mutants display upregulation of the LpoB and PBP1B (MrcB) proteins.

**FIG 1:**
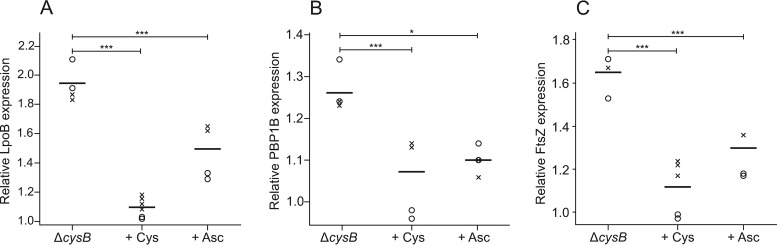
Relative levels (compared to wild-type strain DA5438) of proteins LpoB, PBP1B, and FtsZ in the Δ*cysB* strain DA28439, grown without and with 0.75 mM cysteine (Cys) or 10 mM ascorbic acid (Asc). Circles and crosses signify identical experiments performed at different times. (A) The relative levels of the LpoB protein are significantly different when the Δ*cysB* strain is grown with cysteine or ascorbic acid (with *P* values of 9.6 × 10^−6^ and 1.8 × 10^−5^, respectively). (B) The relative levels of the PBP1B protein are significantly different when the Δ*cysB* strain is grown with cysteine or ascorbic acid (with *P* values of 3.1 × 10^−3^ and 0.02, respectively). (C) The relative levels of the FtsZ protein are significantly different when the Δ*cysB* strain is grown with cysteine or ascorbic acid (with *P* values of 6.0 × 10^−5^ and 8.0 × 10^−4^, respectively).

The test described above showed that deletion of either PBP1B or LpoB was sufficient to revert the Mec^r^ phenotype of the Δ*cysB* strain to susceptibility. To study how gene expression is altered in the Δ*cysB* mutant (strain DA28439), compared to the wild-type strain (strain DA5438), we examined the global protein expression pattern in bacteria grown with and without cysteine. The expression levels of more than 450 proteins were changed by 50% to 200% in the Δ*cysB* mutant, compared to the wild-type strain. Importantly, the proteomics showed significant increases in the LpoB and PBP1B (MrcB) levels (1.9- and 1.3-fold increases, respectively) in the Δ*cysB* strain, compared to the wild-type strain, during growth in Mueller-Hinton agar (MHA) with glucose (Fig. 1A and B). When the medium was supplemented with 0.3 mM cysteine, the levels of both LpoB and MrcB were reduced to wild-type levels (Fig. 1A and B). A 1.7-fold increase in FtsZ levels was also observed in the Δ*cysB* mutant proteomic analysis and, as with LpoB and PBP1B, the levels reverted to wild-type levels with cysteine treatment (Fig. 1C). Overexpression of FtsZ was shown previously to overcome the essentiality of PBP2, which is one of the two key features in the acquisition of mecillinam resistance in bacteria (13–16, 22).

Interestingly, the level of the stringent-response-stimulated protein RpoS was also increased 6.4-fold in the Δ*cysB* mutant, compared to the wild-type strain. RpoS is positively regulated by the stringent response, which is known to confer mecillinam resistance. Thus, we constructed a Δ*rpoS* Δ*cysB* strain and a Δ*relA* Δ*spoT* Δ*cysB* strain and examined their MICs. The Δ*rpoS* mutant was constructed by transducing the *rpoS*::FRT-Kan-FRT from the Keio 3327 strain. The Δ*relA* Δ*spoT* Δ*cysB* strain was constructed by introducing a resistance cassette into first *relA* and then *spoT* by using Lambda Red recombineering; these cassettes were then flipped out and *cysB*::FRT-Cam-FRT was introduced into the strain by transduction. The two mutant strains, Δ*rpoS* Δ*cysB* and Δ*relA* Δ*spoT* Δ*cysB*, still maintained the Mec^r^ phenotype (MICs of 24 and 32 mg/liter, respectively), indicating that the *cysB* Mec^r^ mechanism is independent of both RpoS and the stringent response (as shown previously by Costa and Antón [21]).

### Overexpression of either LpoB or PBP1B alone is sufficient to increase mecillinam MIC.

Based on the protein analysis, we hypothesized that, in the Δ*cysB* mutant, there was upregulation of a protein (or proteins) that could perform the function of the mecillinam-inactivated PBP2. Two candidate proteins were PBP1B (MrcB) and the PBP1B activator LpoB. Both of these proteins showed increased levels in the Δ*cysB* mutant and reduced levels when cysteine was added, indicating a correlation with the resistance phenotype (i.e., increased PBP1B and LpoB levels correspond to resistance, and wild-type PBP1B and LpoB levels correspond to susceptibility). PBP1B (MrcB) is a class A PBP (aPBP) that has both glycosyltransferase and TPase activities (26). The aPBPs have been suggested to have an important function in the Mec^r^ mechanism (10). LpoB is an activating cofactor of PBP1B (27, 28).

To test this idea, we overexpressed PBP1B (MrcB) and LpoB individually by constructing chromosomal duplications that included only the *lpoB* or *mcrB* gene, and we determined whether susceptibility was reduced. The *lpoB* and *mrcB* genes were duplicated in the wild-type strain (DA5438) by Lambda Red recombineering, resulting in strains with two copies of *lpoB* (strain DA55581) or two copies of *mrcB* (strain DA58001), respectively (Fig. S1). The reason for performing the overexpression test using duplications is that the levels are expected to only double, avoiding potential deleterious effects, as often seen (see below) when overexpression is achieved with a plasmid-borne gene copy. The MICs of mecillinam for the strains with the increased gene dosage were determined in Mueller-Hinton broth (MHB) and MHB with Cys (Table 1). Duplication of *lpoB* alone increased the MIC 192-fold (from 0.125 mg/liter to 24 mg/liter), and duplication of *mrcB* alone increased the MIC 8-fold (from 0.125 mg/liter to 1 mg/liter). When grown on MHA plates supplemented with cysteine, the strains carrying the duplications, DA55581 (*lpoB*) and DA58001 (*mrcB*), retained their respective MICs. (Table 1). The MIC for DA55581 (*lpoB* duplication) is over the mecillinam clinical breakpoint of 8 mg/liter, but the MIC for DA58001 (*mrcB* duplication) is not. We cannot at present explain the relatively larger effect of duplication of LpoB, compared to duplication of PBP1b. We also attempted to overexpress LpoB by cloning the *lpoB* gene on an expression plasmid, but this approach generated only strains containing plasmids with frameshift mutations in the *lpoB* gene, suggesting that high levels of LpoB are lethal.

### Reducing conditions decrease LpoB, PBP1B, and FtsZ levels and revert *cysB* mutants to mecillinam susceptibility.

The proteomic analysis indicated that the Δ*cysB* mutant exhibited an oxidative-stress-like response, which potentially could be involved in mecillinam resistance. The proteins KatE, YfcG, TcyL (FliY), OsmC, YghU, YqjD, SodC, YfcF, GstB, LuxS, MsrA, QorA, GstA, MsrC, YaiA, ArtI, YghA, TcyN (YecC), YbjC, SufA, IscR, AroF, YdcL, LpxC, SufC, FadI (YfcY), ArcA, ThiJ (YajL), ZnuA, IscS, DsbB, and YdhF (24, 29–39) were among the most highly overexpressed (about 2- to 8-fold increases, compared to the wild-type levels) (Table 2). Oxidative stress responses in E. coli are usually mediated through either the *soxRS* or *oxyR* systems (26, 28), but the pattern of upregulation of oxidative stress proteins seen in the Δ*cysB* mutant is not typical of either of the two.

**TABLE 2:** Relative levels of oxidative-stress-associated proteins in the Δ*cysB* mutant DA28439 during growth in MHB and MHB supplemented with 10 mM ascorbic acid or 0.3 mM cysteine

Protein	Protein function	Relative protein level (mean ± standard deviation)^*a*^		
		Δ*cysB*	+Asc	+Cys
KatE	Catalase HPII	8.32 ± 2.61	5.06 ± 1.49	1.46 ± 0.51
YfcG	Disulfide bond oxidoreductase	6.25 ± 0.18	4.01 ± 0.85	1.14 ± 0.11
TcyJ (FliY)	Periplasmic l-cystine-binding protein	5 ± 0.41	2.75 ± 0.98	0.76 ± 0.08
OsmC	Peroxiredoxin	4.95 ± 1.79	2.2 ± 0.61	0.85 ± 0.12
YghU	Disulfide bond oxidoreductase	4.46 ± 0.27	2.89 ± 0.52	1.18 ± 0.24
YqjD	Inner membrane protein associated with ribosomes	4.33 ± 0.14	2.34 ± 0.84	1.01 ± 0.2
SodC	Superoxide dismutase	4.1 ± 1.5	2.11 ± 0.83	0.63 ± 0.09
YfcF	Glutathione *S*-transferase	4.07 ± 0.86	3.03 ± 0.78	1.21 ± 0.28
GstB	Glutathione *S*-transferase	3.78 ± 0.27	2.68 ± 0.59	1.36 ± 0.35
LuxS	*S*-Ribosylhomocysteine lyase	3.59 ± 0.52	2.55 ± 0.67	1.29 ± 0.25
MsrA	Peptide methionine sulfoxide reductase	3.47 ± 0.25	2.98 ± 1.29	1.06 ± 0.1
QorA	Putative quinone oxidoreductase 1	3.36 ± 0.39	2.01 ± 0.61	0.90 ± 0.07
GstA	Glutathione *S*-transferase	3.18 ± 0.38	1.61 ± 0.17	1.1 ± 0.16
MsrC	Free methionine-(*R*)-sulfoxide reductase	3.1 ± 0.39	2.46 ± 0.26	1.35 ± 0.28
YaiA	Hypothetical protein (homologous to hydroquinone dioxygenase in lactobacilli)	2.94 ± 0.14	2.63 ± 0.12	1 ± 0.2
ArtI	Putative periplasmic ABC transporter protein	2.93 ± 0.32	2.91 ± 1.09	0.85 ± 0.12
YghA	NADP^+^-dependent aldehyde reductase	2.85 ± 0.15	1.98 ± 0.68	0.72 ± 0.11
TcyN (YecC)	Cystine/cysteine ABC transporter ATP-binding subunit	2.81 ± 0.17	2.17 ± 0.56	1.15 ± 0.17
YbjC	Uncharacterized protein YbjC	2.80 ± 0.06	1.97 ± 0.4	1.29 ± 0.11
SufA	Iron-sulfur cluster insertion protein	2.67 ± 0.22	3.27 ± 0.05	1.44 ± 0.18
IscR	2Fe-2S DNA-binding transcriptional repressor	2.31 ± 0.25	1.78 ± 0.11	1.36 ± 0.1
AroF	2-Dehydro-3-deoxyphosphoheptonate aldolase	2.19 ± 0.01	1.56 ± 0.29	1.35 ± 0.02
YdcL	Putative lipoprotein	2.15 ± 0.12	1.4 ± 0.14	0.93 ± 0.11
LpxC	UDP-3-*O*-acyl-*N*-acetylglucosamine deacetylase	2.11 ± 0.28	1.48 ± 0.17	1.18 ± 0.19
SufC	Component of SufBCD Fe-S cluster scaffold complex	2.01 ± 0.14	2.41 ± 0.24	1.27 ± 0.41
FadI (YfcY)	3-Ketoacyl-coenzyme A thiolase	1.96 ± 0.03	1.35 ± 0.2	0.98 ± 0.04
ArcA	DNA-binding transcriptional dual regulator	1.94 ± 0.09	1.52 ± 0.23	1.08 ± 0.09
ThiJ (YajL)	Chaperone protecting proteins in response to oxidative stress	1.94 ± 0.1	1.39 ± 0.12	1.19 ± 0.14
ZnuA	Periplasmic Zn^2+^-ABC transporter protein	1.91 ± 0.02	1.37 ± 0.26	0.96 ± 0.08
IscS	Cysteine desulfurase	1.89 ± 0.08	1.99 ± 0.27	1.31 ± 0.11
DsbB	Disulfide bond formation protein	1.88 ± 0.05	1.32 ± 0.03	1.14 ± 0.05
YdhF	Putative oxidoreductase	1.78 ± 0.2	1.41 ± 0.22	1.04 ± 0.07

a Measured proteins levels were compared to the levels in the wild-type strain (strain DA5438), which was set to 1 for each protein. Asc, ascorbic acid; Cys, cysteine.

If the *cysB* mutants experience oxidative stress and this is associated with resistance, it would be expected that reducing agents could reverse the mutant to susceptibility. To examine this idea, we performed MIC assays with the Δ*cysB* mutant grown on medium supplemented with several reducing agents. Addition of either cysteine, cystine, dithiothreitol (DTT), glutathione, or ascorbic acid (16, 40–42) decreased the MIC of mecillinam from 32 mg/liter to 0.38, 0.125, 0.38, 0.38, or 0.75 mg/liter, respectively (Table 3).

**TABLE 3:** MICs of mecillinam in the wild-type strain (strain DA5438) and the Δ*cysB* mutant (strain DA28439) during growth on medium supplemented with cystine or the reductants cysteine, DTT, glutathione, or ascorbic acid, as well as during anaerobic respiration

Growth condition	Mecillinam MIC (mg/liter)	
	DA5438 (wild-type)	DA28439 (Δ*cysB*)
MHB	0.125	32
MHB + cysteine	0.19	0.38
MHB + cystine	0.19	0.125
MHB + DTT	0.094	0.38
MHB + glutathione	0.19	0.38
MHB + ascorbic acid	0.125	0.75
Anaerobic respiration	0.125	0.064

In addition, the proteomics showed that the levels of LpoB, PBP1B, and FtsZ in the Δ*cysB* mutant were decreased to wild-type levels when the growth medium was supplemented with cysteine or ascorbic acid (Fig. 1). The interpretation of these findings is that resistance in the Δ*cysB* mutant is caused by an oxidative-stress-like response that can be reverted to susceptibility by reducing agents. Further support for this comes from the observation that growth during anaerobic respiration also reduces the MIC of mecillinam in the Δ*cysB* mutant to the wild-type level (Table 3).

To elucidate whether any of the known regulators of oxidative stress responses was involved in conferring mecillinam resistance, we performed the following test. We deleted known redox regulators (*soxS*, *soxR*, *oxyR*, *arcA*, *arcB*, *fnr*, and *ahpC* genes) in the wild-type strain and the Δ*cysB* mutant and examined the effect on the MIC of mecillinam (Table S5). If a regulatory system acts as a repressor in the wild-type strain, then its inactivation would be expected to result in mecillinam resistance. Conversely, if it acts as an activator system in the Δ*cysB* mutant, then its inactivation would be expected to result in loss of resistance. None of these predictions was observed. Thus, deletion of these regulators in the wild-type background caused no increase in the MIC of mecillinam. Similarly, deletion of the regulators in the Δ*cysB* mutant conferred no reduction in the MIC of mecillinam. These results indicate that the mecillinam resistance we observe in the Δ*cysB* mutant is independent of the function of any known redox regulator. It can be noted, however, that inactivation of the *soxR*, *arcA*, and *arcB* genes increased the MIC, which might indicate that the cells experienced even greater oxidative stress.

Furthermore, we tried inducing an oxidative stress response in wild-type E. coli MG1655 (DA5438) by using the oxidizing agents H_2_O_2_ and atrazine (34, 43), to determine whether this would have an effect on the mecillinam susceptibility of the bacteria. The MICs of mecillinam were determined by broth dilution in MHB at concentrations of H_2_O_2_ and atrazine known to induce oxidative stress in E. coli (44, 45). However, the MIC of mecillinam for the wild-type strain was 0.125 mg/liter irrespective of whether H_2_O_2_ and atrazine were present.

## DISCUSSION

**FIG 2:**
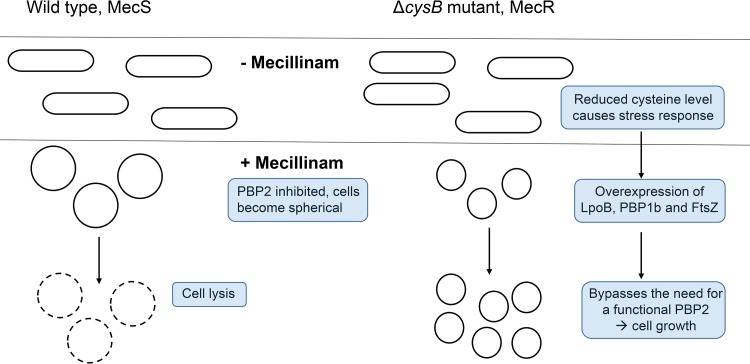
Model for how *cysB* mutations confer mecillinam resistance. In a Δ*cysB* mutant, the cysteine level is reduced and, as a result, the cysteine/cystine shuttle system (24, 47) cannot provide the periplasm with enough reducing agents, which results in a stress response that changes the expression levels of >450 proteins, including increases in LpoB and PBP1B levels. The increases in LpoB and PBP1B levels allow cell wall biosynthesis via an alternative pathway, bypassing the need for functional PBP2. Microscopy studies of the wild-type strain and the *cysB* mutant, with and without mecillinam, show that both strains are rod-shaped in the absence of mecillinam and spherical in the presence of mecillinam, with the difference being that the loss of functional PBP2 through mecillinam inhibition is not lethal in *cysB* mutants.

Our previous work showed that inactivation of the cysteine biosynthesis regulator CysB is the major cause of clinical mecillinam resistance, being the only type of Mec^r^ mutation found in a collection of MecR mutants isolated at different places in Sweden over several months, as well as in a number of isolates from other European countries (11). Here we present data that show that, for Δ*cysB* mutations to confer mecillinam resistance in E. coli, upregulation of PBP1B (MrcB) and its activator LpoB is needed and the upregulation appears to be dependent on the redox state of the cell. Based on the reported results, we propose the following model for the mechanism of mecillinam resistance (Fig. 2). (i) In a wild-type E. coli strain, inhibition of PBP2 by mecillinam is lethal due to inactivation of the elongasome machinery and the β-lactam-induced futile cycle of production of un-cross-linked glycans and their subsequent rapid degradation by Slt (as described by Cho et al. [22]). (ii) In a *cysB* mutant, the absence of CysB prevents cysteine biosynthesis, resulting in an atypical oxidative stress response due to the reduced cysteine levels. (iii) The stress results in induction of expression of PBP1B, LpoB, and FtsZ. (iv) Overexpression of FtsZ renders PBP2 and the Rod system nonessential, and increased levels of LpoB and PBP1B can end the futile toxic cycle of PG synthesis and degradation performed by the Mec-targeted Rod system and Slt.

Our presented data are compatible with this model. Thus, the *cysB* mutant shows increased expression of several proteins known to be overproduced during oxidative stress responses in the cell. Furthermore, by adding various reducing agents (e.g., cysteine, DTT, glutathione, and ascorbic acid) to a resistant *cysB* mutant, the resistance can be reverted to full susceptibility, indicating that resistance is associated with redox conditions. This idea is supported by a previous study by Hufnagel et al., which suggested that *cys* (in that case, *cysE*) mutants could owe their Mec^r^ to hyperoxidation of the cell (46). A reasonable explanation for why reduced cysteine levels cause oxidative stress is that the cysteine/cystine shuttle system located in the inner membrane of E. coli uses cysteine to remove periplasmic H_2_O_2_ (24, 47) and thus reduced cysteine levels are expected to reduce scavenging of H_2_O_2_. In addition, we previously showed in growth studies that the concentrations of reductants (specifically cysteine) in human urine are high enough (75 μM) to partly or fully reverse the Mec^r^ phenotype of *cysB* mutants (48).

Interestingly the proposed oxidative stress response and associated Mec^r^ are not mediated through any of the known oxidative stress response regulators, as deletion of *soxS*, *soxR*, *oxyR*, *arcA*, *arcB*, *ahpC*, or *fnr* in the wild-type strain or the Δ*cysB* mutant did not influence Mec^r^ levels. In addition, RpoS is known to regulate oxidative stress protection genes, but the deletion of *rpoS* similarly had no effect on the MIC of the wild-type strain or the Δ*cysB* mutant. In line with this, growing wild-type E. coli with the oxidants H_2_O_2_ and atrazine did not affect the susceptibility of the strain to mecillinam. Thus, at present the genetic basis for the upregulation is unknown.

Among the upregulated proteins in the Δ*cysB* mutant were PBP1B, LpoB, and FtsZ. Overexpression of FtsZ is a known key factor in overcoming the adverse effects of mecillinam (16). The involvement of PBP1B and LpoB in resistance was established by the observations that (i) overexpression of either *mrcB* or *lpoB* alone in a wild-type strain conferred an increase in the mecillinam MIC and (ii) inactivation of either the *mrcB* or *lpoB* gene made *cysB* mutants susceptible. At present, it is unclear which regulatory system (or systems) is involved in causing the upregulation of the PBP1b, LpoB, and FtsZ proteins in the Δ*cysB* mutant.

The results presented demonstrate that functional PBP1B and LpoB proteins are necessary to confer mecillinam resistance and overexpression of either protein can generate resistance. Thus, our model postulates that PBP1B-LpoB can bypass the need for functional PBP2. Supporting this hypothesis is a recently published study describing the mecillinam resistome (10). Using mutants overexpressing FtsZ (thereby overcoming one of the two antimicrobial effects of mecillinam), Lai et al. selected Mec^r^ mutants and identified a total of 143 loci involved in Mec^r^ (10). They could show that overexpression of MepS and other E. coli PG endopeptidases conferred Mec^r^. They proposed that the Rod complex crippled by mecillinam is saved by the overexpressed endopeptidases stimulating the activity of aPBPs, which work outside the Rod complex and are not affected by mecillinam. A conclusion from their work was that the cleavage of cell wall cross-links by PG endopeptidases results in the activation of PG synthesis by aPBPs. By specifically testing PBP1B (which belongs to class A), they suggested that elevated PG endopeptidase activity stimulates PG synthesis by the aPBPs by increasing either the levels or the activity of aPBPs. These data fit well with our observations of the essentiality of LpoB and PBP1B overexpression in mecillinam resistance conferred by Δ*cysB* mutations. However, we do not see any overproduction of endopeptidases in the proteomics data. Instead, the gene regulatory response caused by the Δ*cysB* mutation increases the levels of LpoB and PBP1B, thereby directly achieving stimulation of PG synthesis.

In conclusion, we propose a model for the clinically relevant *cysB*-mediated Mec^r^, describing how stress caused by lack of cysteine biosynthesis results in upregulation of genes that overcome both of the adverse effects of mecillinam, i.e., (i) the inactivation of PBP2 and (ii) the toxic cycle of glycan production and degradation. These results contribute to the elucidation of mecillinam resistance and show that alterations in the redox state of the bacteria (for example, through exposure to reducing agents such as ascorbic acid) might induce antibiotic susceptibility in a Mec^r^ E. coli strain.

## MATERIALS AND METHODS

### Bacterial strains and media.

All strains used in this study are derivatives of E. coli K-12 MG1655 (DA5438) or strains from the Keio collection (11, 49). All strains are listed in Table S1 in the supplemental material. When not otherwise specified, bacteria were grown in MHB and plated on MHA (Becton, Dickinson and Co.). When appropriate, the medium was supplemented with the antibiotics tetracycline (12.5 mg/liter), chloramphenicol (Cam) (12.5 mg/liter), kanamycin (Kan) (100 mg/liter), or ampicillin (100 mg/liter) (all from Sigma-Aldrich). For MIC determinations, the medium was supplemented with l-cysteine (0.75 mM), cystine (0.75 mM), DTT (10 mM), glutathione (10 mM), or ascorbic acid (10 mM) (all from Sigma-Aldrich). The concentrations of cysteine and cystine were chosen based on our previous studies (11, 48), and the concentrations of glutathione and ascorbic acid was chosen from the study by Goswami et al. (50). DTT was used at the same concentration as glutathione and ascorbic acid. Strains were preserved by freezing overnight cultures in 10% dimethyl sulfoxide at −80°C.

Anaerobic respiration medium contained 1.5% agar (Oxoid), 0.03% casein (Becton, Dickinson and Co.), 100 μM CaCl_2_, 1 mM MgSO_4_, 1× M9 salts, 0.2% glycerol, and 10 mM potassium nitrate. The agar and casein were added to double-distilled water, and then the solution was autoclaved. The other ingredients were prepared as stock solutions, which were sterile filtered and then added to the anaerobic respiration medium. When bacteria were grown on the anaerobic respiration plates, an AnaeroGen patch from Thermo Fisher was placed in the anaerobe jar together with the plates, to achieve an anaerobic environment.

### MIC determinations.

MICs were determined for mecillinam (using MIC test strips from Liofilchem), meropenem (MIC evaluators from Oxoid), ampicillin (Etest strips from bioMérieux), and cefotaxime (Etest strips from bioMérieux). Bacteria were grown overnight in MHB and then were diluted 500-fold in phosphate-buffered saline (PBS) (13 mM phosphate, 137 mM NaCl [pH 7.4]) before being spread evenly on MHA plates (some supplemented with l-cysteine, cystine, DTT, glutathione, or ascorbic acid) and on anaerobe respiration plates. A MIC test strip, Etest, or MIC evaluator was placed on the plates, and the results were analyzed after ∼18 h of incubation at 37°C.

### Broth dilution MICs in medium supplemented with H_2_O_2_ or atrazine.

MICs were determined for mecillinam in MHB containing 0, 0.5, or 2 mM H_2_O_2_ or 0, 0.5, or 0.8 mg/liter atrazine. Overnight cultures were diluted to a 0.5 McFarland standard in MHB with or without H_2_O_2_ or atrazine, and 95-μl aliquots of the diluted cultures were added to wells in 96-well plates with serial 2-fold dilutions of mecillinam; the final concentrations were between 0.064 and 32 mg/liter. The results were read after ∼18 h of incubation at 37°C.

### PCR and Sanger sequencing.

PCRs were performed using either Phusion High-Fidelity DNA polymerase (Thermo Fisher) or DreamTaq (Thermo Fisher), with the following protocol: 95°C (DreamTaq) or 98°C (Phusion) for 5 min; 29 cycles of 95°C (DreamTaq) or 98°C (Phusion) for 30 s, the proper primer annealing temperature for 30 s, and 72°C for the proper period for the sequence of interest (1 min kb^−1^); and 72°C for 7 min before cooling to 4°C. PCR products were purified using the GeneJET PCR purification kit (Thermo Fisher) and then were sequenced by Eurofins MWG Operon (Ebersberg, Germany). Sequences were analyzed using CLC Genomics Workbench software. All PCR primers used are listed in Table S2.

### Strain construction.

Strains with duplications of *lpoB* and *mrcB* (encoding PBP1B and MrcB, respectively) were constructed in the wild-type background with standard genetic techniques in combination with a recently developed method for generating chromosomal duplications, as described by Näsvall et al. (12, 51) (see Fig. S1). In brief, a *cat-sacB* resistance marker was introduced to force a duplication of the region around the gene of interest by temperature-controlled Lambda Red recombineering (8, 13–16, 51–54), using a pSim5-Tet plasmid (55). The strain was subsequently grown on medium supplemented with Cam, to maintain selection for the duplication.

To construct strains with gene deletions, a Cam or Kan cassette was introduced to replace the whole gene, using the same recombineering method as described above. In some cases, phage P1 transduction was used to move a Kan cassette insertion in the gene of interest from the respective Keio strain (49). All PCR primers used for amplification of the resistance cassettes and screening for insertion of the cassettes are listed in Table S2.

### Proteomics.

Overnight cultures were diluted 1:500 in 20 ml MHB with 0.4% glucose, incubated with shaking at 37°C until the optical density at 600 nm was 0.4, and then moved to an ice-water bath for >10 min. The cells were pelleted in a centrifuge (4,500 rpm for 8 min at 4°C), and all traces of medium were removed. Cells were washed three times in 1 ml ice-cold PBS. The procedure was performed in 1.5-ml tubes, pelleting the cells in a centrifuge at maximum speed for 2 min in 4°C. The pellets were frozen and stored at −80°C until analysis. Pellets were sent to the Proteomics Core Facility at the University of Gothenburg for global protein analysis. The samples were lysed and the total protein concentrations in the lysates were measured. Then an aliquot of each sample was reduced, alkylated, and digested with trypsin. The digested peptides were chemically labeled with a tandem mass tag reagent and then combined. The sample was prefractionated by liquid chromatography, and each fraction was analyzed by liquid chromatography-mass spectrometry. In total, 28,000 peptides were identified at a false discovery rate of 1%, yielding ∼2,500 E. coli proteins quantified across the samples. A more detailed description of the proteomics analysis is available in the supplemental material.

The mass spectrometry raw data and the search results have been deposited at the ProteomeXchange Consortium via the PRIDE partner repository (56), with the data set identifier PXD007647.

### Statistics.

To test for the significance of the differences in the relative expression of LpoB, PBP1b, and FtsZ in the Δ*cysB* strain DA28439 grown with or without cysteine or ascorbic acid, we used a multiple linear model adjusted for batch effects, with the significance of the effects being evaluated using *t* tests.

## Supplementary Material

Supplemental file 1
