# Supplementary material for: Upregulation of PBP1B and LpoB in cysB Mutants Confers Mecillinam (Amdinocillin) Resistance in Escherichia coli
Source: Antimicrob Agents Chemother. 2019 Sep 23;63(10):e00612-19. doi: 10.1128/AAC.00612-19 (PMC6761508; doi:10.1128/AAC.00612-19)
Supplement: Supplemental file 1 [file AAC.00612-19-s0001.pdf]

Supplementary Data for

Upregulation of PBP1B and LpoB in *cysB* mutants confers mecillinam resistance in *Escherichia coli*

Elisabeth Thulin[a] and Dan I. Andersson[a*]

Supplementary Table 1. List of *E. coli* K12 strains used in this work.

Strain	Genotype	Origin and description
DA5438	*E. coli* MG1655	Lab collection, Wild type
DA28439	*cysB*::FRT-scar	Thulin *et al.* 2015 (1)
DA48398	*relA*::FRT-scar, *spoT*::FRT-scar, *cysB::FRT-Cam-FRT*	This work
DA49479	*cysB*::FRT-scar, *mrcB*::FRT-Kan-FRT	This work
DA49480	*cysB*::FRT-scar, *dacA*::FRT-Kan-FRT	This work
DA49481	*cysB*::FRT-scar, *dacB*::FRT-Kan-FRT	This work
DA49482	*cysB*::FRT-scar, *ampC*::FRT-Kan-FRT	This work
DA49483	*cysB*::FRT-scar, *dacD*::FRT-Kan-FRT	This work
DA49484	*cysB*::FRT-scar, *pbpG*::FRT-Kan-FRT	This work
DA49485	*cysB*::FRT-scar, *yfeW*::FRT-Kan-FRT	This work
DA49486	*cysB*::FRT-scar, *pbpC*::FRT-Kan-FRT	This work
DA49487	*cysB*::FRT-scar, *ampH*::FRT-Kan-FRT	This work
DA49488	*cysB*::FRT-scar, *dacC*::FRT-Kan-FRT	This work
DA49489	*cysB*::FRT-scar, *mrcA*::FRT-Kan-FRT	This work
DA50858	*cysB*::FRT-scar, *lpoB*::FRT-Kan-FRT	This work
DA54878	*cysB*::FRT scar, *mrcB* Cys776Ala	This work
DA55445	*cysB*::FRT scar, *mrcB* Cys794Ala	This work
DA55581	*lpoB* duplicated	This work
DA58001	*mrcB* duplicated	This work
DA61055	*cysB*::FRT-scar, *soxS*::FRT-Kan-FRT	This work
DA61056	*soxS*::FRT-Kan-FRT	This work
DA61057	*cysB*::FRT-scar, *soxR*::FRT-Kan-FRT	This work
DA61058	*soxR*::FRT-Kan-FRT	This work
DA61059	*cysB*::FRT-scar, *oxyR*::FRT-Kan-FRT	This work
DA61060	*oxyR*::FRT-Kan-FRT	This work
DA61061	*cysB*::FRT-scar, *arcA*::FRT-Kan-FRT	This work
DA61062	*arcA*::FRT-Kan-FRT	This work
DA61063	*cysB*::FRT-scar, *arcB*::FRT-Kan-FRT	This work
DA61064	*arcB*::FRT-Kan-FRT	This work

DA61065	*cysB*::FRT-scar, *ahpC*::FRT-Kan-FRT	This work
DA61066	*ahpC*::FRT-Kan-FRT	This work
DA61067	*cysB*::FRT-scar, *fnr*::FRT-Kan-FRT	This work
DA61068	*fnr*::FRT-Kan-FRT	This work
DA61069	*cysB*::FRT-scar, *rpoS*::FRT-Kan-FRT	This work
DA61070	*rpoS*::FRT-Kan-FRT	This work
K24	*fnr*::FRT-Kan-FRT	Keio collection
K52	*soxR*::FRT-Kan-FRT	Keio collection
K53	*soxS*::FRT-Kan-FRT	Keio collection
K179	*arcB*::FRT-Kan-FRT	Keio collection
K196	*arcA*::FRT-Kan-FRT	Keio collection
K303	*mrcB*::FRT-Kan-FRT	Keio collection
K311	*dacA*::FRT-Kan-FRT	Keio collection
K347	*dacB*::FRT-Kan-FRT	Keio collection
K1861	*ahpC*::FRT-Kan-FRT	Keio collection
K2738	*ampC*::FRT-Kan-FRT	Keio collection
K3194	*lpoB*::FRT-Kan-FRT	Keio collection
K3278	*dacD*::FRT-Kan-FRT	Keio collection
K3292	*pbpG*::FRT-Kan-FRT	Keio collection
K3308	*yfeW*::FRT-Kan-FRT	Keio collection
K3327	*rpoS*::FRT-Kan-FRT	Keio collection
K3573	*pbpC*::FRT-Kan-FRT	Keio collection
K3680	*ampH*::FRT-Kan-FRT	Keio collection
K3815	*oxyR*::FRT-Kan-FRT	Keio collection
K3943	*dacC*::FRT-Kan-FRT	Keio collection
K3961	*mrcA*::FRT-Kan-FRT	Keio collection

Supplementary Table 2. PCR primers and oligonucleotides used in this work.

Primer name	Sequence	Used for
cysB FWD	GAAAAAGACGGAAAGGCGA	Screening of *cysB*
cysB REV	GCGAGGCGGGTAATTAGA	Screening of *cysB*
relA KO LiTr FWD	AACCGACGCGCGTCGATAACA TCCGGCACCTGGTTGAGTTTGT AGGCTGGAGCTGCTTC	Knock out of *relA*
relA KO LiTr REV	ATGGTTGCGGTAAGAAGTGCA CATATCAATAAGGCTGGTGCAT ATGAATATCCTCCTTAG	Knock out of *relA*
relA KO scr FWD	CCAACGCTTTACGCTACT	Screening of *relA*
relA KO scr FWD	ATATACCATTGCGCGACT	Screening of *relA*
spoT KO LiTr FWD	TTGTATCTGTTTGAAAGCCTGA ATCAACTGATTCAAACCTTGTA GGCTGGAGCTGCTTC	Knock out of *spoT*

spoT KO LiTr REV	TTAATTTCGGTTTCGGGTGACT TTAATCACGTCTGGCATCCATA TGAATATCCTCCTTAG	Knock out of *spoT*
spoT KO scr FWD	GAAATCGAAGAAGGTCTG	Screening of *spoT*
spoT KO scr REV	CGTTATGAGGTTTGTGGA	Screening of *spoT*
mrcB FWD	TCACCCTAACCCTCTCCCT	Screening of *mrcB*
mrcB REV	CGCGCCATTGGTATATCT	Screening of *mrcB*
dacA FWD	GTACCACTTCAACCACCT	Screening of *dacA*
dacA REV	CATTTATTACTACCGCGCA	Screening of *dacA*
dacB FWD	GGGACCAGAAGCAAAAAAA	Screening of *dacB*
dacB REV	ATCAGGCCTACAAGAGAA	Screening of *dacB*
ampC FWD	GTCGGGTGTCAGGGTTAT	Screening of *ampC*
ampC REV	GCGCGTAACAGTAAAAAAG	Screening of *ampC*
dacD FWD	TACGTTTCTCTTCCTGCT	Screening of *dacD*
dacD REV	TCCGTTATTTCTGCTGTCT	Screening of *dacD*
pbpG FWD	CGTTTTTTGCTACCGCGTT	Screening of *pbpG*
pbpG REV	TTCCGTGACGATCGCTTT	Screening of *pbpG*
yfeW FWD	GCGGGCGGTTTGTTTATC	Screening of *yfeW*
yfeW REV	TATCCGGCCTACGAAATCG	Screening of *yfeW*
pbpC FWD	GCGTTCGGTTTGATGATG	Screening of *pbpC*
pbpC REV	TAACGCTGGTGTATCTGG	Screening of *pbpC*
ampH FWD	CTGGAATGAAGAATTGCGA	Screening of *ampH*
ampH REV	TGGTGAGCAGGAAAAAGA	Screening of *ampH*
dacC FWD	CCGATGGTGAAGTAAAGT	Screening of *dacC*
dacC REV	TGATATTGTGGTGAGCGA	Screening of *dacC*
mrcA FWD	ATCTCCTTATCACCCGTC	Screening of *mrcA*
mrcA REV	TCAGGCTTATGGGGTTTC	Screening of *mrcA*
lpoB FWD	AACGAAAGGCAAGAACCC	Screening of *lpoB*
lpoB REV	GCTGACGCACAACAAAAC	Screening of *lpoB*
lpoB dupl FWD	TTAATAGTGGGCTTAGTGGCGG GAGTTTTCTCATTGAACATGTA GGCTGGAGCTGCTTC	Duplication of *lpoB*
lpoB dupl REV	ACGCTGCGTGGCCTTTCCAGAG GATGCATCTCCAGCCCTCCATA TGAATATCCTCCTTAG	Duplication of *lpoB*
lpoB dupl scr FWD	GTGCAGACGGGCGAAATTAT	Screening of the *lpoB* duplication
lpoB dupl scr REV	GTCATCAAGATTCACCCCTT	Screening of the *lpoB* duplication
mrcB dupl FWD	CTCGTTTACGTTATCATTCACTT TACATCAGAGATATACCTGTAG GCTGGAGCTGCTTC	Duplication of the *mrcB* gene
mrcB dupl REV	CTCACCCCGGCCCTCTCCCACA GGGAGAGGGAGAAAACCACAT ATGAATATCCTCCTTAG	Duplication of the *mrcB* gene
mrcB dupl scr FWD	TGGTAGTAATTAACATCTAA	Screening of the *mrcB* duplication
mrcB dupl scr REV	TCAGGCGCAAAAGCCCGATT	Screening of the *mrcB* duplication
soxS FWD	TGCCAGGGATGGTTCTTT	Screening of *soxS*
soxS REV	GGGGTTAGCAGCGCTTTA	Screening of *soxS*
soxR FWD	ATCTGCCTCTTTTCAGTG	Screening of *soxR*
soxR REV	AGAGAAAGACAAAGACCGGAA A	Screening of *soxR*
oxyR FWD	TCCGTTTCTGTGAGCAAT	Screening of *oxyR*
oxyR REV	ACACCACCTTTAACTACCC	Screening of *oxyR*
arcA FWD	AAAGAAGTTACAACGGACGA	Screening of *arcA*
arcA REV	AAAGTACCCACGACCAAG	Screening of *arcA*
arcB FWD	TATCTTAACTGCGTGCGG	Screening of *arcB*

arcB REV	AAAATCGATGGACGGGAA	Screening of *arcB*
ahpC FWD	GGGGGCCATTTTACTTTT	Screening of *ahpC*
ahpC REV	GCAGCACCCGAAGAATTA	Screening of *ahpC*
fnr FWD	GCCATACAGGGTCTCCTT	Screening of *fnr*
fnr REV	CCTTGCCATCGGGTTATT	Screening of *fnr*
rpoS FWD	TGGGGTTGTCGGTAGCAGA	Screening of *rpoS*
rpoS REV	AGGCGGGGCAAAAAATAG	Screening of *rpoS*

Supplementary Table 3. MICs of mecillinam (measured by Etest) of wild type, Δ*cysB* mutant and Δ*cysB*, Δ*pbp* double mutants. The different Δ*pbp* mutations were transduced by phage P1 from relevant strains in the Keio collection.

Strain	Genotype	PBP deleted	Keio strain	Mec MIC (mg/L)
DA5438	*E. coli* wild type	-	-	0.125
DA28439	*E. coli* Δ*cysB*	-	-	32
DA49479	*E. coli* Δ*cysB*, Δ*mrcB*	PBP1b	K303	0.125
DA49480	*E. coli* Δ*cysB*, Δ*dacA*	PBP5	K311	32
DA49481	*E. coli* Δ*cysB*, Δ*dacB*	PBP4	K347	64
DA49482	*E. coli* Δ*cysB*, Δ*ampC*	AmpC	K2738	48
DA49483	*E. coli* Δ*cysB*, Δ*dacD*	PBP6b	K3278	48
DA49484	*E. coli* Δ*cysB*, Δ*pbpG*	PBP7	K3292	96
DA49485	*E. coli* Δ*cysB*, Δ*yfeW*	PBP4b	K3308	96
DA49486	*E. coli* Δ*cysB*, Δ*pbpC*	PBP1c	K3573	64
DA49487	*E. coli* Δ*cysB*, Δ*ampH*	AmpH	K3680	64
DA49488	*E. coli* Δ*cysB*, Δ*dacC*	PBP6a	K3943	192
DA49489	*E. coli* Δ*cysB* Δ*mrcA*	PBP1a	K3961	96
DA50858	*E. coli* Δ*cysB*, Δ*lpoB*	PBP1b activator	K3194	0.125

Supplementary Table 4. MICs of mecillinam, meropenem, cefotaxime and ampicillin of the strains DA28696, K303 and K3194.

		MIC (mg/L)			
**Strain**	**Genotype**	mecillinam	meropenem	cefotaxime	ampicillin
DA28696	*E. coli* vild type	0.19	0.03	0.047	3
DA28439	*E. coli* Δ*cysB*	32	0.064	0.047	2
K303	*E. coli* Δ*mrcB*	0.125	0.03	0.032	1.5
K3194	*E. coli* Δ*lpoB*	0.125	0.03	0.047	2

Supplementary Table 5. MICs of mecillinam (measured by MIC test strips) of wild type, Δ*cysB* mutant and Δ*cysB*, redox-regulator deletion double mutants. The different redox-regulator deletions were transduced by phage P1 from relevant strains in the Keio collection to the DA5438 and DA28439 strains.

Strain	Genotype	Keio strain	Mec MIC (mg/L)
DA5438	*E. coli* wild type	-	0.125
DA28439	*E. coli* Δ*cysB*	-	32
DA61055	*E. coli* Δ*cysB*, Δ*soxS*	K53	48
DA61056	*E. coli* Δ*soxS*	K53	0.19

DA61057	*E. coli* Δ*cysB*, Δ*soxR*	K52	>256
DA61058	*E. coli* Δ*soxR*	K52	0.25
DA61059	*E. coli* Δ*cysB*, Δ*oxyR*	K3815	32
DA61060	*E. coli* Δ*oxyR*	K3815	0.25
DA61061	*E. coli* Δ*cysB*, Δ*arcA*	K196	>256
DA61062	*E. coli* Δ*arcA*	K196	0.125
DA61063	*E. coli* Δ*cysB*, Δ*arcB*	K179	>256
DA61064	*E. coli* Δ*arcB*	K179	0.125
DA61065	*E. coli* Δ*cysB*, Δ*ahpC*	K1861	48
DA61066	*E. coli* Δ*ahpC*	K1861	0.19
DA61067	*E. coli* Δ*cysB* Δ*fnr*	K24	32
DA61068	*E. coli* Δ*fnr*	K24	0.125

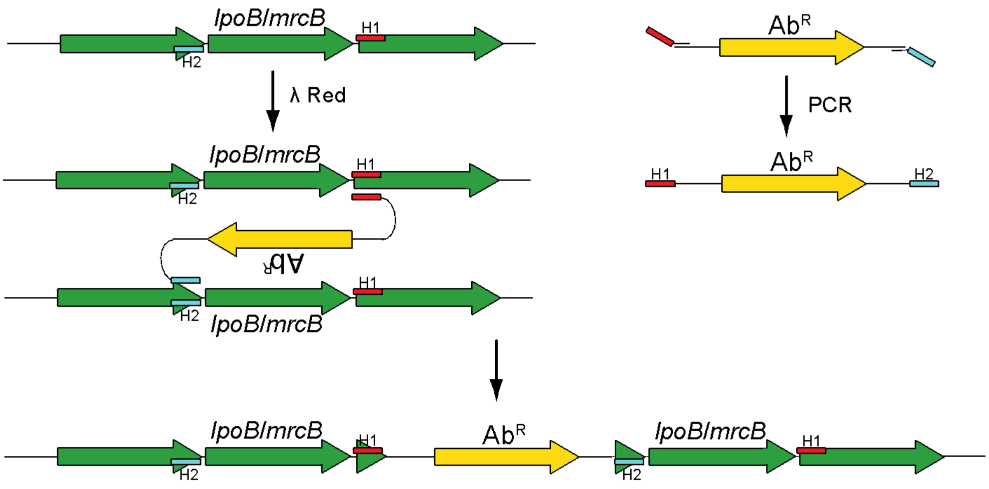

Supplementary Figure 1. The method used for duplication of the *lpoB* and *mrcB* genes, as described by Näsvall *et al.* (2). The *lpoB* and *mrcB* duplications were made by Duplication-Insertion Recombineering, which is a fast and scar-free λ Red recombineering method for efficient transfer of mutations in bacteria developed by Näsvall *et al.* 2016. In the method, transient selection markers are used to transfer mutations between bacterial strains. The method allows for construction of strains with several mutations without any unwanted sequence changes. The method uses λ Red to generate a marker-held tandem duplication, termed Duplication-Insertion (Dup-In). The Dup-Ins are then transferred between strains by transduction and are subsequently lost by homologous recombination, leaving no scar sequence or antibiotic resistance cassette behind.

Detailed proteomics method

Cell Lysis

The samples were homogenized in 250 µl lysis buffer (50 mM Triethylammonium bicarbonate (TEAB; Fluka, Sigma Aldrich) and 2% Sodium dodecyl sulfate (SDS)) with 1 mm silica beads using FastPrep®-24 instrument (MP Biomedicals, OH). The beads were centrifuged at maximum speed for 15 min, the beads were washed with the lysis buffer and centrifuged at maximum speed again. The protein extracts were combined and protein concentration was determined using Pierce™ BCA Protein Assay (Thermo Scientific) and the Benchmark Plus microplate reader (BIO-RAD) with BSA solutions as standards.

Protein Digestion and Labeling

Aliquots containing 50 or 100 µg of each sample were digested with trypsin using the filter-aided sample preparation (FASP) method (3). Briefly, protein samples were reduced with 100 mM dithiothreitol at 60°C for 30 min, transferred on 10 kDa MWCO Nanosep centrifugal filters (Pall Life Sciences, Ann Arbor, USA), wahed with 8M urea solution and alkylated with 10 mM methyl methanethiosulfonate in 50 mM TEAB and 1% sodium deoxycholate. Digestion was performed in 50 mM TEAB, 1% sodium deoxycholate at 37°C in two stages: the samples were incubated with 500 or 1000 ng (trypsin:protein ratio 1:100) of Pierce MS-grade trypsin (Thermo Scientific) for 3h, then 500 or 1000 ng more of trypsin was added and the digestion was performed overnight. The peptides were collected by centrifugation, labelled using TMT 10-plex isobaric mass tagging reagents (Thermo Scientific) according to the manufacturer instructions. The labeled samples were mixed, sodium deoxycholate was removed by acidification with 10% TFA.

The combined labeled sample was fractionated using either strong cation exchange chromatography (SCX) or basic reversed-phase chromatography (bRP-LC). SCX was performed on the AKTA chromatography system (GE Healthcare Life Sciences, Sweden) using PolySULFOETHYL A™ column (100x2.1mm, 5µm 300Å, PolyLC Inc., Columbia, USA) at 0.25 ml/min and a gradient from 0 to 20% B in 20 min, to 40% B in 10 min and to 100% B in 10 min; solvent A was 25 mM ammonium formate, pH 2.8 and solvent B was 500 mM ammonium formate, pH 2.8. Eighteen peptide-containing fractions were desalted using PepClean C18 spin columns (Thermo Fisher Scientific) according to the manufacturer's guidelines, the purified eluates were dried on Speedvac, the dried fractions were reconstituted in 3% acetonitrile, 0.2% formic acid for analysis.

The bRP-LC was performed on the Dionex Ultimate 3000 UPLC system (Thermo Fischer Scientific) using the Waters XBridge BEH C18 column (3.0x150 mm, 3.5µm, Waters Corporation, Milford, USA) and the gradient from 3% to 40% solvent B over 17 min, from 40% to 100% B over 5 min, 100% B for 5 min, all at the flowrate of 0.4 ml/min; solvent A was 10 mM ammonium formate in water at pH 10.00, solvent B was 90% acetonitrile, 10% 10 mM ammonium formate in water at pH 10.00. The initial 20 fraction were combined into 8 pooled fractions in the order 5+13, 6+14, 7+15 etc. The pooled fractions were dried on Speedvac and reconstituted in 3% acetonitrile, 0.2% formic acid for analysis.

LC-MS/MS Analysis

Each SCX fraction was analyzed on Q Exactive mass spectrometer interfaced with Easy-nLC 1000 nanoflow liquid chromatography system (both - Thermo Fisher

Scientific). Peptides were trapped on the in-house packed trap column (75 µm X 4.5 cm) and separated on the in-house packed analytical column (75 µm X 20 cm) packed with 3 µm Reprosil-Pur C18-AQ particles (Dr. Maisch GmbH, Ammerbuch, Germany). The following gradient was run at 200 nl/min; 7-27 % B-solvent (98% acetonitrile with 0.2% formic acid) over 70 min, 27-40 % B over 5 min, 40-80 % B over 5 min with a final hold at 80 % B for 10 min, using 0.2% formic acid in water as solvent A. Precursor ion scans were performed in a scan range of m/z 400-1600 at 70 000 resolution setting. MS/MS analysis was performed in a data-dependent mode, with the top ten most abundant doubly or multiply charged precursor ions in each MS scan selected for fragmentation by stepped high energy collision dissociation (stepped HCD) of NCE-value of 30, 40 and 50. The MS2 scans were performed at 35000 resolution (at m/z 200), AGC 1e5 with a maximal injection time of 110 ms, a fixed first mass of m/z 100, an isolation window of 1.6 Da, intensity threshold of 1.1e4 and a dynamic exclusion of 30 seconds.
Each bRP-LC fraction was analyzed on Orbitrap Fusion Tribrid mass spectrometer interfaced with Easy-nLC 1000 nanoflow liquid chromatography system (both - Thermo Fisher Scientific). Peptides were trapped on the Acclaim Pepmap 100 C18 trap column (100 µm X 2 cm, particle size 5 µm, Thermo Fischer Scientific) and separated on the in-house packed C18 analytical column (75 µm X 30 cm, particle size 3 µm) using the gradient from 5% to 32% B in 75 min, from 32% to 100% B in 5 min, solvent A was 0.2% formic acid and solvent B was 80% acetonitrile, 0.2% formic acid. Precursor ion mass spectra were recorded at 120 000 resolution, the most intense precursor ions were selected ('top speed' setting with a duty cycle of 3s), fragmented using CID at collision energy setting of 35, spectra and the MS/MS spectra were recorded in ion trap with the maximum injection time of 40 ms and the isolation window of 0.7 Da. Charge states 2 to 7 were selected for fragmentation, dynamic exclusion was set to 45 s with 10 ppm tolerance. MS3 spectra for reporter ion quantitation were recorded at 50 000 resolution with HCD fragmentation at collision energy of 60 using the synchronous precursor selection of the 7 most abundant MS/MS fragments, with the maximum injection time of 100 ms.

Database Search and Quantification
Data analysis was performed using Proteome Discoverer version 1.4 or version 2.1 (Thermo Fisher Scientific). The SCX data was processed with Proteome Discoverer 1.4 using the Swissprot database (November 2014) with Escherichia coli taxonomy filter. Mascot 2.3.2 (Matrix Science) was used as a search engine with precursor mass tolerance of 5 ppm and fragment mass tolerance of 0.2 Da. No missed cleavages were accepted, mono-oxidation on methionine was set as a variable modification, methylthiolation on cysteine and TMT-6 reagent modification on lysine and peptide N-terminus were set as a fixed modification. The target-decoy approach was used for the filtering of the identifications, with the target false discovery rate of 1% as a threshold to filter confident peptide IDs. Reporter ion intensities were quantified in MS2 spectra at 0.02 Da mass tolerance. The resulting ratios were normalized in the Proteome Discoverer 1.4 on the median protein value of 1.0 in each sample.
The bRP-LC data was processed with Proteome Discoverer 2.1 using Swissprot database for *Escherichia coli* K12 (May 2016), supplemented with the mutant sequences and common proteomic contaminants. Mascot 2.5.1 (Matrix Science) was used as a search engine with precursor mass tolerance of 10 ppm and fragment mass tolerance of 0.5 Da. One missed cleavage was accepted, mono-oxidation on methionine was set as a variable modification, methylthiolation on cysteine and TMT-6 reagent

modification on lysine and peptide N-terminus were set as a fixed modification. Percolator was used for the validation of identification results, target false discovery rate of 1% was used as a threshold to filter confident peptide identifications. Reporter ion intensities were quantified in MS3 spectra at 0.003 Da mass tolerance. The resulting ratios were normalized in the Proteome Discoverer 2.1 on the median protein value of 1.0 in each sample.

References

1. **Thulin E, Sundqvist M, Andersson DI.** 2015. Amdinocillin (mecillinam) resistance mutations in clinical isolates and laboratory-selected mutants of Escherichia coli. Antimicrob Agents Chemother **59**: 1718–1727.

2. **Näsvall J, Knöppel A, Andersson DI.** 2016. Duplication-Insertion Recombineering: a fast and scar-free method for efficient transfer of multiple mutations in bacteria. Nucl Acids Res **45**: e33–3.

3. **Wisniewski JR, Zougman A, Nagaraj N, Mann M**. 2009. Universal sample preparation method for proteome analysis. Nat Methods **6**:359-362.
